# Temperature-dependent gating pathways in TRPV3

**DOI:** 10.1038/s41598-026-44194-0

**Published:** 2026-03-26

**Authors:** Guangyu Wang

**Affiliations:** 1 Department of Physiology and Membrane Biology, University of California School of Medicine, Davis, CA USA; 2 Department of Drug Research and Development, Institute of Biophysical Medico-Chemistry, Reno, NV USA

**Keywords:** Biochemistry, Biological techniques, Biophysics, Biotechnology, Chemical biology, Computational biology and bioinformatics, Neuroscience, Physiology, Structural biology, Systems biology, Chemistry, Mathematics and computing

## Abstract

Both hot and cold sensations of the homotetrameric thermosensitive transient receptor potential vanilloid 1–4 (TRPV1-4) channels have been predicted by a single Gibbs–Helmholtz equation for a change in molar heat capacity. However, the heat capacity model has not been examined for TRPV3 channels involving inactivation. Given the mirrored heat and cold sensitivity in TRPV1 with a shared starter, the same case should be detected for TRPV3. To test this hypothesis, the temperature-dependent quaternary and tertiary structures of oxidized TRPV3 in the presence and absence of the natural cannabinoid tetrahydrocannabivarin (THCV) at the active vanilloid site were characterized along a lipid-dependent gating pathway. Further thermoring analyses showed that gating state-dependent thermostability allowed oxidized TRPV3 to be activated and then inactivated by THCV only below 30 °C. However, no inactivation would be observed above 30 °C once the lipid at the active vanilloid site was released by THCV binding. More importantly, despite the distinct tertiary and quaternary structures in cold- and heat-evoked open states, the initial cold activation of oxidized TRPV3 still shared a similar thermosensitivity with heat activation. Therefore, such two temperature-dependent gating pathways of oxidized TRPV3 actually still resulted from symmetric cold and heat activation, supporting the heat capacity model, regardless of the subsequent inactivation. In contrast, the initial inactivation resulted in unpredictable pore dilation along with a tetramer-to-pentamer transition.

**Supplementary Information:**

The online version contains supplementary material available at 10.1038/s41598-026-44194-0.

Heat-responsive homotetrameric thermosensitive transient receptor potential vanilloid 1–4 (TRPV1-4) channels have a specific activation threshold and a high temperature sensitivity Q_10_, which is the ratio of activation rates or open probabilities (P_o_) of an ion channel measured at two temperatures 10 °C apart^1–8^. A recent study revealed that reduced or oxidized mouse TRPV3 (mTRPV3) in the absence or presence of the C612-C619 disulfide bond can be activated by heat-induced unfolding of the least-stable K614-N647 H-bond in the pore domain (PD) (S5-S6) or the least-stable R416-D519 H-bond at the interface between the pre-S1 domain and voltage sensor-like domain (VSLD) (S1-S4) at the specific melting temperature threshold (T_m,th_) of 52 °C or 40 °C, respectively. Furthermore, the specific temperature coefficient or sensitivity Q_10_ of mTRPV3 is closely related to a change in molar heat capacity (ΔC_p_). For a gating transition from a reduced and closed state to an oxidized and open state, ΔC_p_ is about 8.68 kcal/mol-K. This value decreases to 0.762 kcal/mol-K for an oxidized closed mTRPV3^9^. In this case, based on a single Gibbs–Helmholtz equation, both heat and cold unfolding-induced channel activations of TRPV3 should be observed^10^. However, the latter activation may not be directly detected if it occurs at a temperature below the freezing point of water^11^.

Previous studies have shown that capsaicin-induced release of a phosphatidylinositol (PI) lipid from an active vanilloid site decreases the heat activation threshold of TRPV1^12–14^. Therefore, when the phosphatidylcholine (PC) lipid at the corresponding vanilloid site is outcompeted by a ligand such as the natural cannabinoid tetrahydrocannabivarin (THCV), TRPV3 is expected to be activated above the freezing point of water. Given that both cold and heat activations of the minimal thermosensitive rat TRPV1 (rTRPV1) channel without the unstructured pore turret (604–626) upon the PI release from the same active vanilloid site share a similar structural or functional temperature coefficient (Ω_10_ or Q_10_, respectively)^15^, the same case should be observed for TRPV3 if both cold and heat activations start from the PC release from the same active vanilloid site.

To test this hypothesis, cryogenic electron microscopy (cryo-EM) structures of oxidized human TRPV3 (hTRPV3) activated by THCV at 4 °C were compared with those of heat-activated oxidized mTRPV3 at 42 °C^16,17^. In addition, since the removal of the unstructured pore turret (604–626) does not significantly affect the heat activation of rTRPV1^8,18^, the cryo-EM structures of rTRPV1 in the PI-bound closed state at 4 °C, the PI-free activated state at 4 °C and 42 °C were used as positive controls. After identifying two different temperature-dependent quaternary structures in the cold- and heat-evoked open states, the thermoring structures of THCV-gated oxidized hTRPV3 at 4 °C were characterized using a highly sensitive thermodynamic tool developed and examined in several thermosensitive proteins^9,13–15,19–28^, and then compared with those of heat-gated oxidized mTRPV3 at 42 °C. As inactivation is involved in both heat activation of rTRPV1 and cold activation of hTRPV3^14,17^, cryo-EM structures of homotetrameric inactivated and homopentameric pore-dilated hTRPV3 at 4 °C were also analyzed.

The results showed that the two open states of oxidized hTRPV3 or mTRPV3 at low and high temperatures had similar thermosensitivity but distinct quaternary and tertiary thermoring structures for different thermostabilities. This led to inactivation below 30 °C and no inactivation above 30 °C even in the presence of THCV at the active vanilloid site. These temperature-dependent gating pathways demonstrated that even if subsequent inactivation was involved, the initial hot and cold sensing abilities of the thermosensitive TRPV3 channel can still be linked and explained by the single Gibbs–Helmholtz equation for a significant ΔC_p_, supporting the heat capacity model. In contrast, the initial inactivation in homotetrameric oxidized hTRPV3 induced unpredictable pore dilation in pentameric oxidized hTRPV3.

## Results

### Asymmetric intersubunit interactions of rTRPV1 during cold and heat activations

**Fig. 1:**
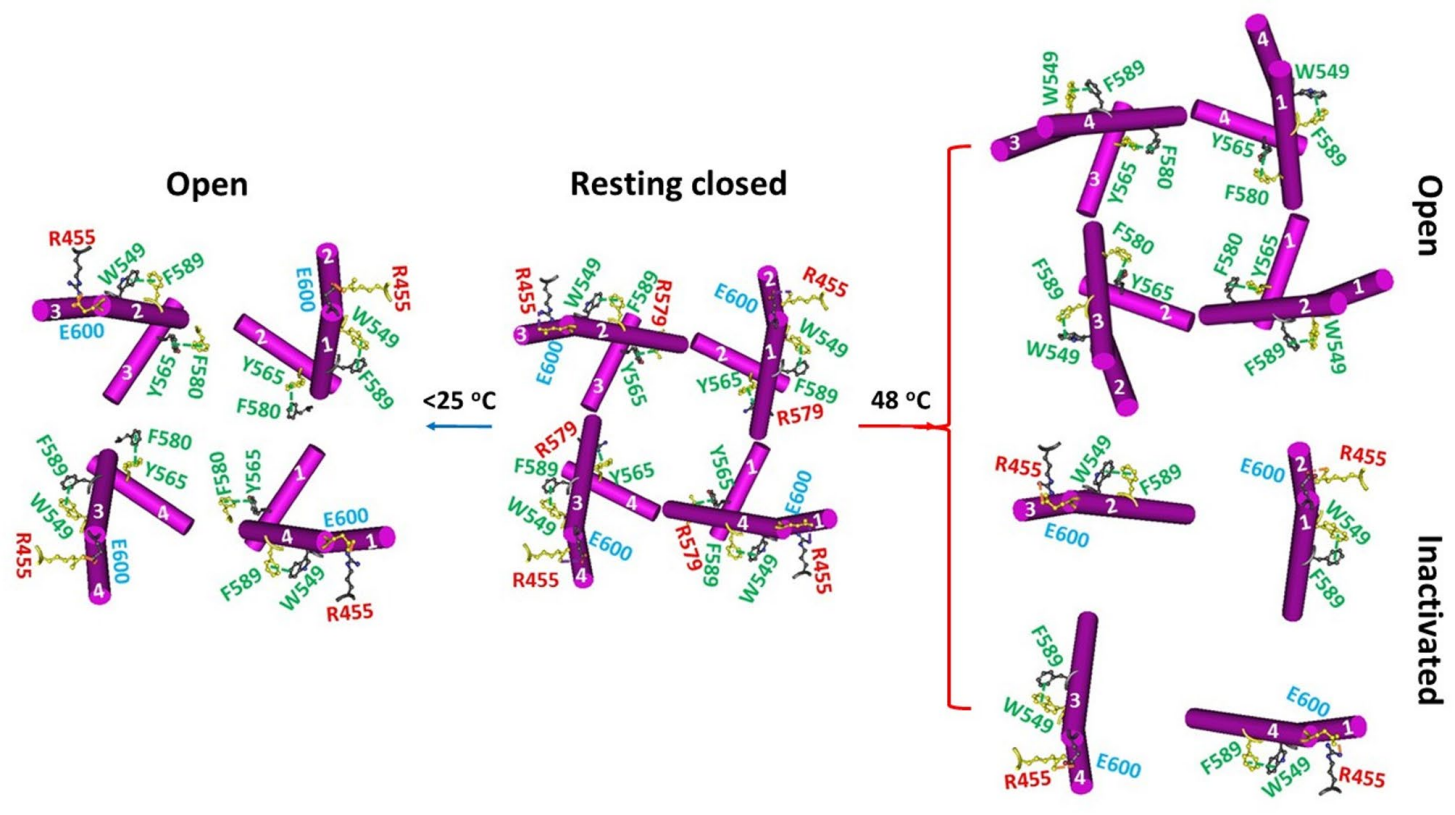
Intersubunit interactions in reduced rTRPV1 during channel gating. The homo-tetrameric cryo-EM structures of the reduced rTRPV1 channel in MSP2N2 nanodiscs in the resting closed, activated and inactivated states at 4 and 42 °C (PDB ID, 7LP9, 8U3L, 7LPE and 7LPD, respectively) were utilized for the model.

Previous studies have shown that the release of PI from the active vanilloid site is required for the activation of homotetrameric rTRPV1 by heat^12,13^. Therefore, it is important to investigate whether any intersubunit interactions change during heat activation. In the PI-bound resting closed state at 4 °C, R579 in the PD of one subunit formed a cation-π interaction with Y565’ in the S4-S5 linker from a neighboring subunit (PDB: 7LP9). These swapping R579-Y565’ cation-π interactions may be crucial for homotetrameric channel closure, as the equivalent Y602-R616 cation-π interactions in TRPV4 promote the closed state^29^. Additionally, E600 in the PD formed a salt bridge with R455’ in the VSLD of another neighboring subunit. Finally, the W549-F589’ swapping π interactions were also observed at the PD/VSLD’ interfaces (PDB: 7LP9) (Fig. 1)^12^.

When PI at the vanilloid site was displaced by capsaicin at 48 °C, the Y565’-R579 π interactions were replaced by Y565’-F580 π interactions and the E600-R455’ bridges were broken in the open state (PDB: 7LPE). In contrast, the Y565’-R579 π bridges were disrupted but the E600-R455’ bridges remained in the inactivated state (PDB: 7LPD) (Fig. 1)^12–14^. On the other hand, although the same intersubunit interactions were present in the open state along with an exchange between resiniferatoxin (RTx) and PI at the vanilloid site at 4 °C (7RQW)^30^, the hybrid swapping interactions as shown in the heat-evoked open and inactivated states appeared in the RTx-free open state below 25 °C (8U3J or 8U3L) (Fig. 1)^12–14,31^. Thus, despite mirrored cold and heat sensitivity upon PI release from the same vanilloid site, both tertiary and quaternary structures were different during cold and heat activations from the same starter^15^.

### Heat-evoked channel opening at 42 °C rearranges the intersubunit interactions of oxidized mTRPV3

**Fig. 2:**
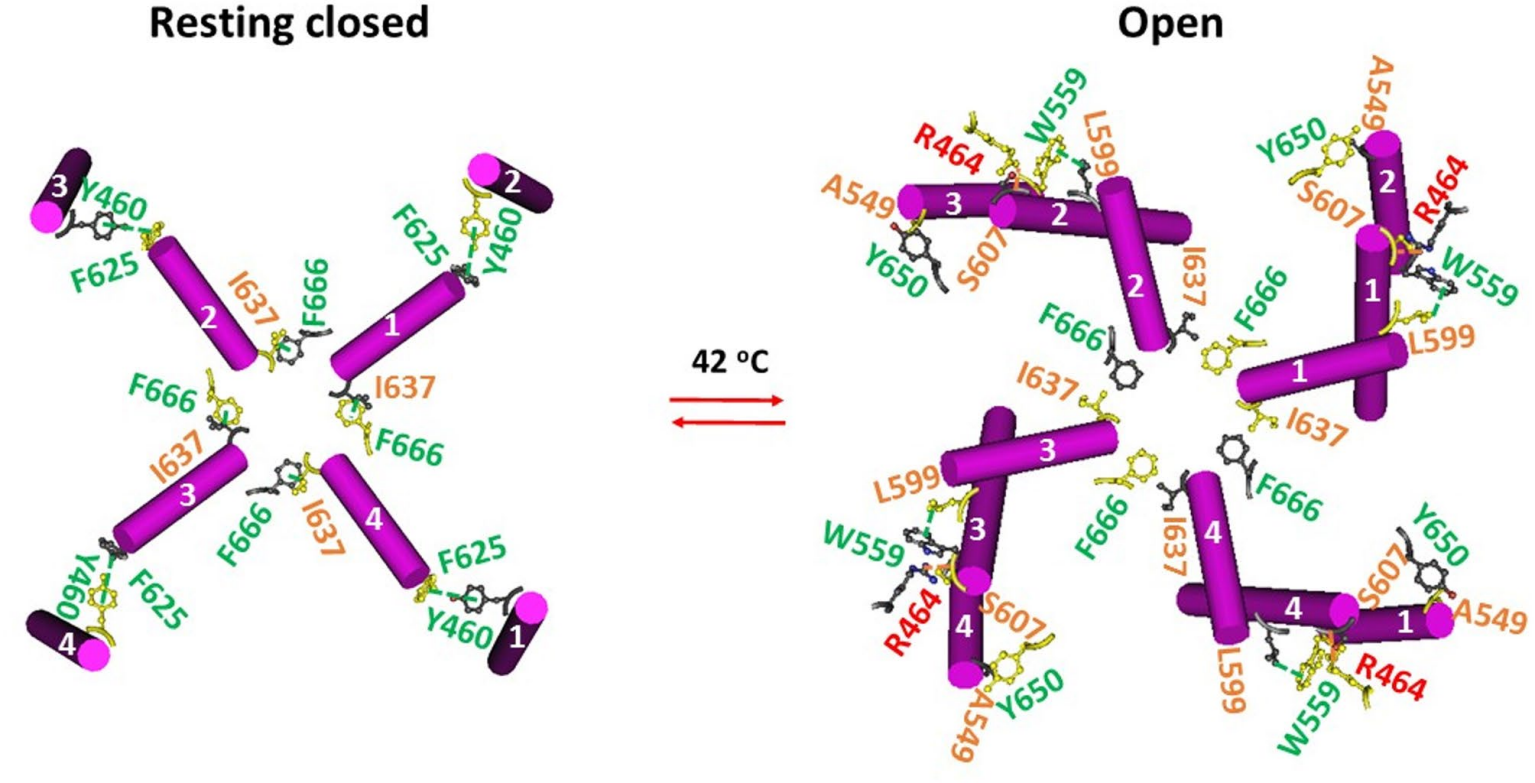
Intersubunit interactions in oxidized mTRPV3 during heat-induced opening. The homo-tetrameric cryo-EM structures of the oxidized mTRPV3 channel in cNW11 nanodiscs in the closed state at 4 °C and the open state at 42 °C (PDB ID, 7MIM and 7MIO, respectively) were used for the model.

In the presence of the PC lipid at the active vanilloid site of the closed homotetrameric mTRPV3 channel with the C612-C619 disulfide bonds in cNW11 nanodiscs at 4 °C, two critical intersubunit interactions were identified along the PC-dependent minimal gating pathway from D396 to K705^9^. One was a π–π interaction between F625 from the pore helix of the nth subunit and Y460’ from the VSLD of the (n + 1)th subunit; the other was a CH-π interaction between F666 from the PD of the nth subunit and I637’ from the pore helix of the (n + 1) subunit. Thus, each PD was linked with the neighboring VSLD and PD (Fig. 2)^16^.

In contrast, in the heat-evoked open state at 42 °C, those swapping interactions mentioned above were replaced by the swapping L599-W559’ and A549-Y650’ π interactions, as well as the intersubunit R464-S607’ H-bonds (Fig. 2)^16^. Hence, the quaternary structure underwent significant changes during channel opening at high temperature (Fig. 2).

### THCV-evoked channel gating transitions at 4 °C rearrange the intersubunit interactions of oxidized hTRPV3 differently

**Fig. 3:**
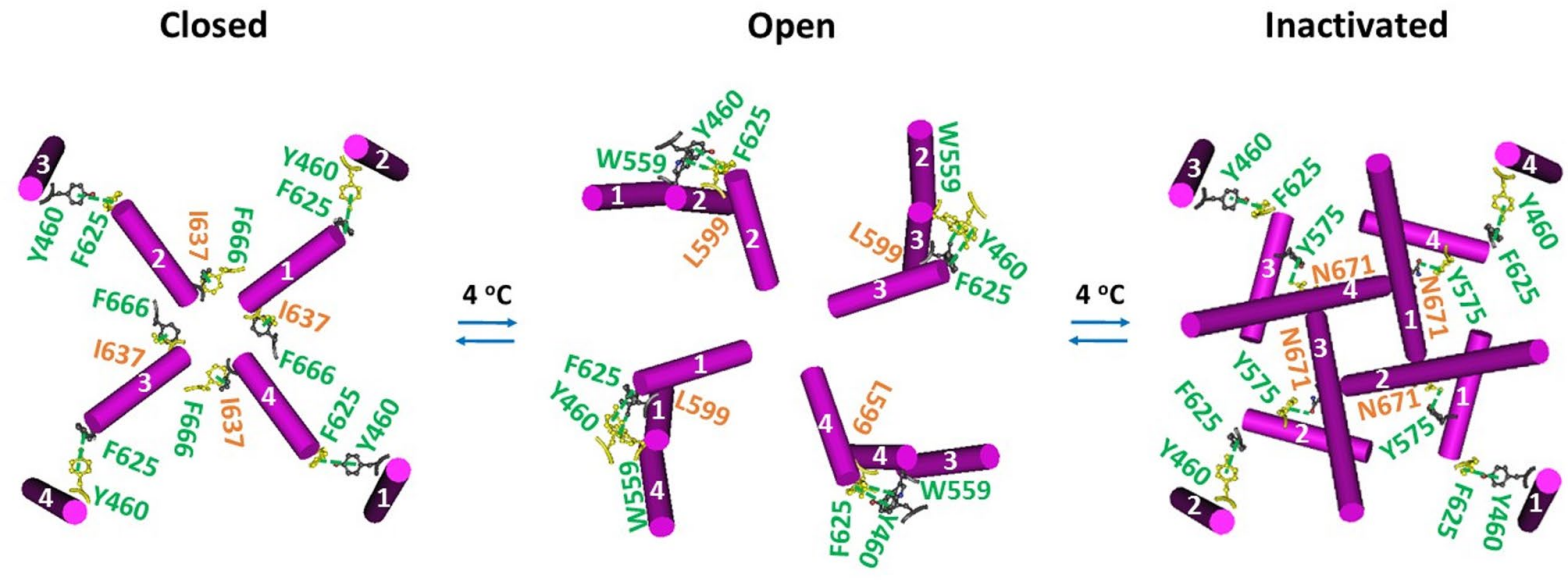
Intersubunit interactions in oxidized hTRPV3 during cold-induced channel gating transitions. The homo-tetrameric cryo-EM structures of the hTRPV3 channel with THCV bound in cNW30 nanodiscs in the closed, open and inactivated states at 4 °C (PDB ID, 8V6K, 8V6L and 8V6M, respectively) were used for the model.

The same intersubunit interactions were observed in the oxidized closed hTRPV3 channel in cNW30 nanodiscs at 4 °C (Fig. 3). When THCV outcompeted the PC lipid from the active vanilloid site, along with the intact Y460-F625’ intersubunit interactions serving as an anchor, the I637-F666’ swapping π interactions were replaced by the swapping L599-W559’ π interactions in an open state or by N671-Y575’ π interactions in an inactivated state (Fig. 3)^17^. Therefore, THCV-induced gating transitions of oxidized hTRPV3 at 4 °C were completely different from those of oxidized mTRPV3 at 42 °C, even starting from the same closed state.

### Closed oxidized hTRPV3 in cNW30 nanodiscs at 4 °C is unstable in the absence of THCV

**Fig. 4:**
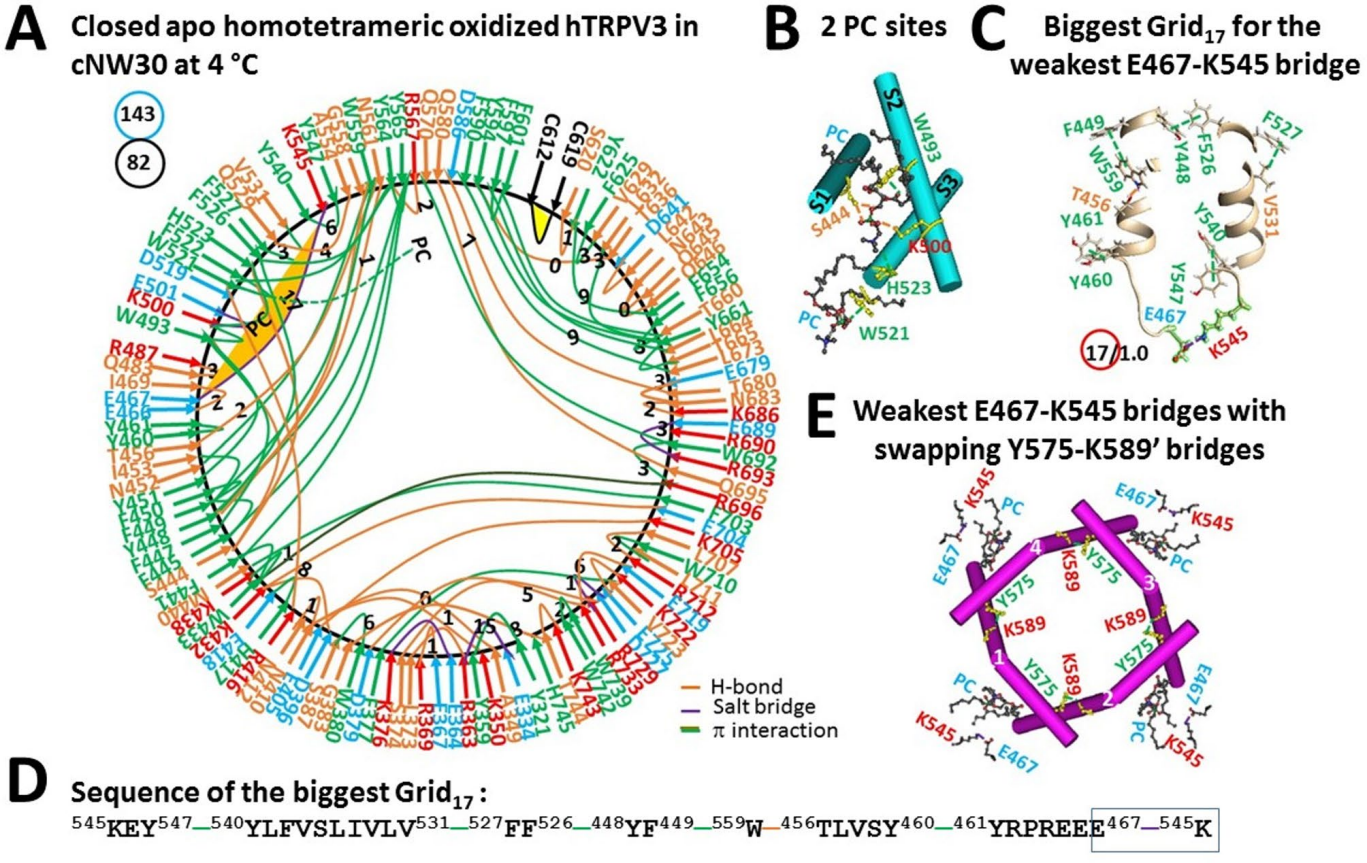
Thermoring structures of apo closed and oxidized hTRPV3 in cNW30 at low temperature. (**A**) The grid-like noncovalently interacting mesh network along the PC-dependent gating pathway of a single subunit of oxidized and closed hTRPV3 in the absence of THCV but with cNW30 nanodics at 4 °C (PDB: 8V6K). Salt bridges, π interactions, and H-bonds between paired amino acid side chains along the PC-dependent gating pathway from K318 to G754 are denoted in purple, green, and orange, respectively. The specific constrained grid sizes necessary to regulate the least-stable noncovalent interactions in the grids are denoted with black numbers. The identified weakest E467-K545 salt bridge in the biggest Grid_17_ is emphasized in orange. The total grid sizes and the total grid size-controlled noncovalent interactions along the PC-dependent gating pathway are displayed in cyan and black circles, respectively. (**B**) Two PC sites in the VSLD and at the vanilloid site. (**C**) The structure of the biggest Grid_17_ with a 17-residue size to regulate the weakest E467-K545 salt bridge in the VSLD. (**D**) The sequence of the biggest Grid_17_ to control the weakest E467-K545 salt bridge highlighted in the blue box. (**E**) Allosteric** g**ating coupling between the weakest intrasubunit E467-K545 salt bridges and the Y575-K589’ swapping interactions near the lower gate in the closed state, along with the PC lipids at the vanilloid sites.

Oxidized closed hTRPV3 in cNW30 nanodiscs at 4 °C exhibited some thermoring structures similar to oxidized closed mTRPV3 in cNW11 nanodiscs at 42 °C^9^. However, the distinct PC binding at the active vanilloid site resulted in some differences along the PC-dependent minimal gating pathway from D396 to K705. In cNW11 nanodiscs, one PC lipid in the VSLD is linked by S444 and K500 and the other at the vanilloid site is sandwiched by W521 and Q695^9^. In contrast, PC in the VSLD was packaged by S444, W493, K500 and H523 but PC at the vanilloid site only attached to W521 in cNW30 nanodiscs (Fig. 4A, B)^17^. In addition, the E610-N647-K614-E610 bridges in the PD of closed oxidized mTRPV3 at 42 °C were absent in closed oxidized hTRPV3 at 4 °C. Finally, the Q570-W692-R696 π interactions were absent in closed oxidized mTRPV3 at 42 °C but present in closed oxidized hTRPV3 at 4 °C (Fig. 4A)^9^. In this case, in order to understand the temperature-dependent gating pathways of oxidized TRPV3 upon a ligand exchange, it is necessary to evaluate the thermal stability of TRPV3 in different gating states. This can be achieved by using the grid thermoring model that has been recently developed and examined^9,13–15,19–28^.

To accomplish this, the tertiary structure of the TRPV3 subunit was depicted as a systematic grid-like mesh network of noncovalent interactions. Once this network was constrained by graph theory, each noncovalent bridge was found to correspond to the shortest cyclic path, known as a grid or a thermoring. In addition to the direct path represented by the bridge, other nearby noncovalent bridges and segments of the polypeptide chain could create the shortest reverse path. The minimum number of free and unreactive amino acid side chains involved in this process was defined as the grid size (s). By assigning a unique grid size to each noncovalent bridge to symbolize the minimum folding energy, the energy landscape of the tertiary folding of TRPV3 could be visualized using this grid thermodynamic model so that the biggest grid could be identified to control the weakest or least-stable noncovalent bridge and denoted as “Grid_s_”.

When the gating pathway was extended from D396 to K318 in the N-terminal and from K705 to G754 in the C-terminal, the total numbers of grid sizes and tertiary noncovalent interactions were 143 and 82, respectively (Fig. 4A). Thus, their ratio, referred to as the systematic thermal instability (T_i_), was 1.74, higher than the 1.21 of closed oxidized mTRPV3 in cNW11 nanodiscs at 42 °C (Table 1)^9^.

**Table 1:** Comparison of local cold-induced thermoring structural changes of hTRPV3 along the PC-dependent gating pathway from K318 to G754.

PDB ID	8GKA	9DIJ	8V6K	8V6L	8V6M
Construct	hTRPV3				
Lipid at the active vanilloid site	PC	free	PC	THCV	
Redox state	Oxidized				
Lipid environment			cNW30		
Sampling temperature, °C	4				
Gating state	Inactivated	Pore-dilated	Closed	Open	Inactivated
# of the biggest Grid_s_	Grid_9_	Grid_11_	Grid_17_	Grid_16_	Grid_18_
grid size (s)	9	11	17	16	18
# of energetically equivalent basic H-bonds (n) controlled by Grid_s_	1.2	0.8	1.0	1.0	1.9
Total non-covalent interactions (N)	97	75	82	82	73
Total grid sizes (S), a.a	94	121	143	137	114
Calculated T_m,th_ °C	**48**	**40**	**30**	**32**	**37**
Systemic thermal instability (T_i_)	**0.97**	**1.61**	**1.74**	**1.67**	**1.56**
Calculated Ω_10_,_cold_ at E = 1 kcal/mol				**3.0**	
Experimental Q_10,heat_				**2.32**	
Experimental T_th_, °C			**33**		
Refs for T_th_ and Q_10_			(3, 9, 16, 32)	(9, 32)	

The comparative parameters are highlighted in bold.

Notably, the least-stable R416-D519 salt bridge in the biggest Grid_17_ of closed oxidized mTRPV3 in cNW11 nanodiscs at 42 °C was accompanied by the H471-Y540-Y547-H471 π interactions^9^. In contrast, the same R416-D519 salt bridge of closed oxidized hTRPV3 in cNW30 nanodiscs at 4 °C was present along with the least-stable E467-K545 salt bridge controlled by the new biggest Grid_17_ in the VSLD (Fig. 4A, C)^17^. It had a 17-residue thermoring from K545 to Y547, Y540, V531, F527, F526, Y448, F449, W559, T456, Y460, Y461, E467 and back to K545 (Fig. 4D). With 1.0 basic H-bond energetically equivalent to this least-stable E467-K545 salt bridge at the external interface between S1-S2 and S3-S4 linkers, the calculated T_m,th_ for its heat unfolding was about 30 °C (Table 1), which was similar to the measured slow warm activation threshold of 33 °C of TRPV3 but lower than the calculated T_m,th_ of 40 °C of the oxidized closed mTRPV3 channel in cNW11 nanodiscs at 42 °C^3,9,16,32^. Therefore, oxidized hTRPV3 in cNW30 nanodiscs can remain closed until above 30 °C. Of special note, the same critical swapping Y575-K589’ π interactions as shown in reduced closed hTRPV3 (PDB: 6UW4) were observed between the S4-S5 linkers and the PDs for homotetrameric channel closure (Fig. 4E)^15,33^.

### THCV binding stabilizes oxidized hTRPV3 in the open state at 4 °C

**Fig. 5:**
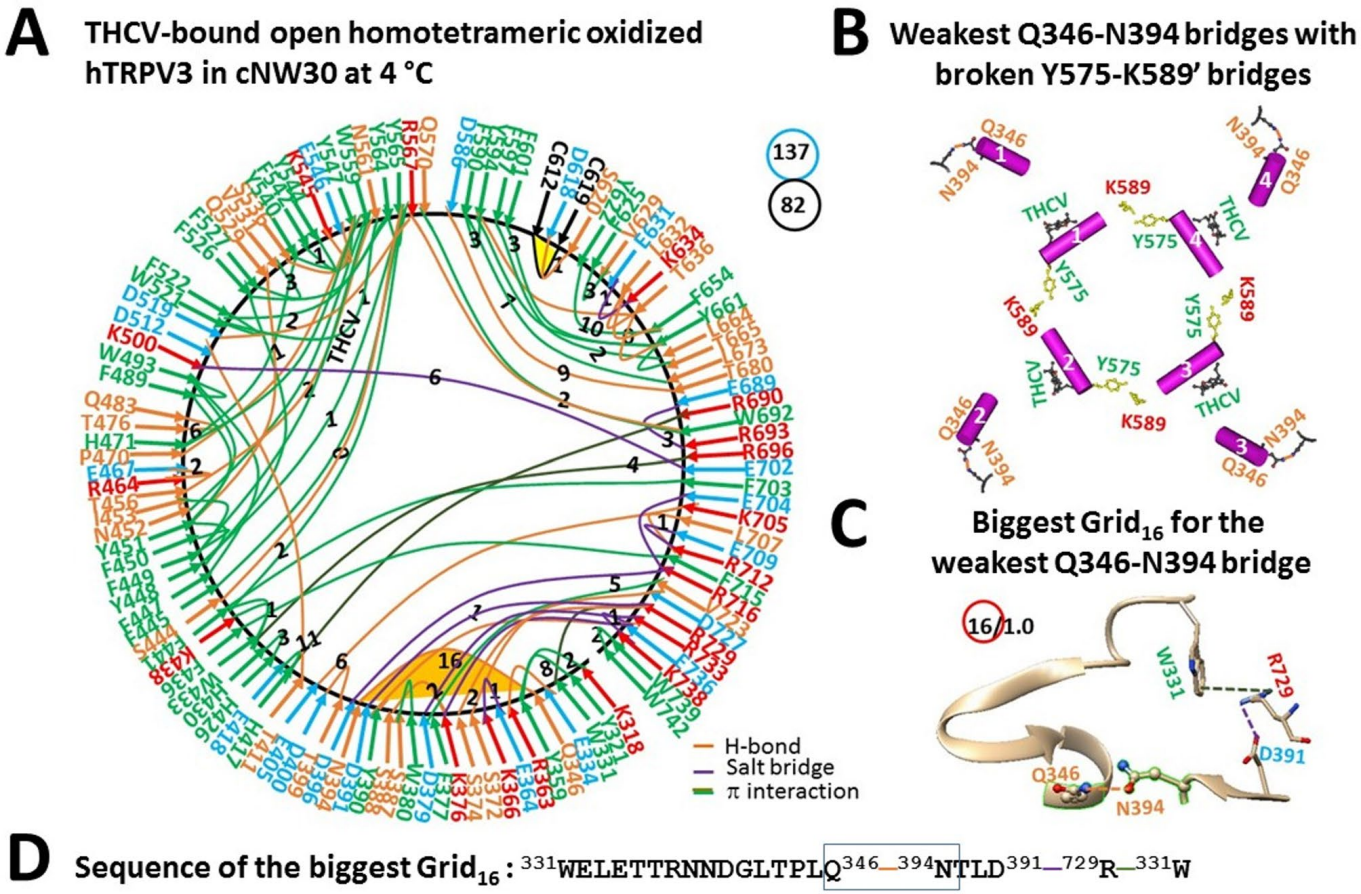
Thermoring structures of open and oxidized hTRPV3 with THCV bound at low temperature. (**A**) The grid-like noncovalently interacting mesh network along the PC-dependent gating pathway of a single subunit of oxidized and open hTRPV3 in the presence of THCV and cNW30 at 4 °C (PDB: 8V6L). Salt bridges, π interactions, and H-bonds between paired amino acid side chains along the PC-dependent gating pathway from K318 to G754 are denoted in purple, green, and orange, respectively. The specific constrained grid sizes necessary to regulate the least-stable noncovalent interactions in the grids are indicated with black numbers. The identified weakest Q346-N394 H-bond in the biggest Grid_16_ is emphasized in orange. The total grid sizes and the total grid size-controlled noncovalent interactions along the PC-dependent gating pathway are displayed in cyan and black circles, respectively. (**B**) Disrupting the E467-K545 bridges disconnected the Y575-K589’ swapping interactions near the lower gate for channel opening by cold, along with the new weakest Q346-N394 bridges and THCV at the vanilloid sites. (**C**) The structure of the biggest Grid_16_ with a 16-residue size to regulate the weakest Q346-N394 H-bond at the NLD/ARD interface. (**D**) The sequence of the biggest Grid_16_ to control the weakest Q346-N394 H-bond highlighted in the blue box.

The closed oxidized hTRPV3 channel in cNW30 nanodiscs was unstable when the PC lipid at the active vanilloid site only attached to W521. When THCV outcompeted the PC lipid from the active vanilloid site at 4 °C, it was wrapped by W521, F522 and N561 via two π interactions and an H-bond. In addition, as expected for mirrored cold and heat activations^10,15^, the E467-K545 salt bridge in the biggest Grid_17_ was disrupted along with the broken Y575-K589’ swapping bridges (Fig. 5A, B)^17^. Although the stimulatory D519-R567 H-bond in the open state of oxidized mTRPV3 in cNW11 nanodiscs at 42 °C was also present in the open oxidized hTRPV3 in cNW30 nanodiscs at 4 °C^9,17^, the presence of the new K500-E702, E405-K705, T411-D512, Y451-Q529 and other bridges allowed the new least-stable Q346-N394 H-bond to be controlled by the biggest Grid_16_ for channel opening in cNW30 nanodiscs at 4 °C (Fig. 5A)^17^. It featured a 16-residue thermoring from W331 to Q346, N394, D391, R729, and back to W331 (Fig. 5C, D). With a basic H-bond energetically equivalent to the least-stable Q346-N394 bridge at the internal interface between the N-terminal linker domain (NLD) and ARD (Fig. 5C), the calculated T_m,th_ to unfold it was about 32 °C (Table 1). In contrast, the different least-stable N412-D512 H-bond is governed by the biggest Grid_23_ for T_m,th_ of 23 °C of the open K169A mutant^15^. Thus, despite the difference in the tertiary thermoring structure between open oxidized hTRPV3 in cNW30 nanodiscs at 4 °C and open oxidized mTRPV3 in cNW11 nanodiscs at 42 °C (Fig. 5A)^9^, the replacement of PC with THCV at the active vanilloid site slightly stabilized the cold-evoked open state of oxidized hTRPV3 in cNW30 nanodiscs.

In that case, although the cold-induced structural transition of globular proteins like ubiquitin and Trp-cage miniprotein below water’s freezing point is non-cooperative, fragmented and locally limited at the protein-water interface^11,34^, a global conformational change during THCV-induced cold activation at low temperature above the freezing point of water was cooperative (Figs. 4A, 5A). Along the same PC-dependent gating pathway from K318 to G754, such a change decreased the total number of grid sizes from 143 to 137 (Figs. 4A, 5A). Thus the systematic thermal instability (T_i_) was 1.67, similar to the 1.74 of closed oxidized hTRPV3 but lower than the T_i_ of 2.55 in the open K169A mutant (Table 1)^15^. Notably, after comparing a change in the total grid sizes and noncovalent bridges between closed and open states at low temperatures, the structural cold sensitivity (Ω_10,cold_) was calculated as 3.0., similar to the heat sensitivity (Q_10,heat_ = 2.32) of mTRPV3^9^. Therefore, the removal of PC from the same active vanilloid site did trigger both cold activation by THCV and heat activation of oxidized TRPV3^10,15^.

### Inactivation further stabilizes oxidized hTRPV3 at 4 °C

**Fig. 6:**
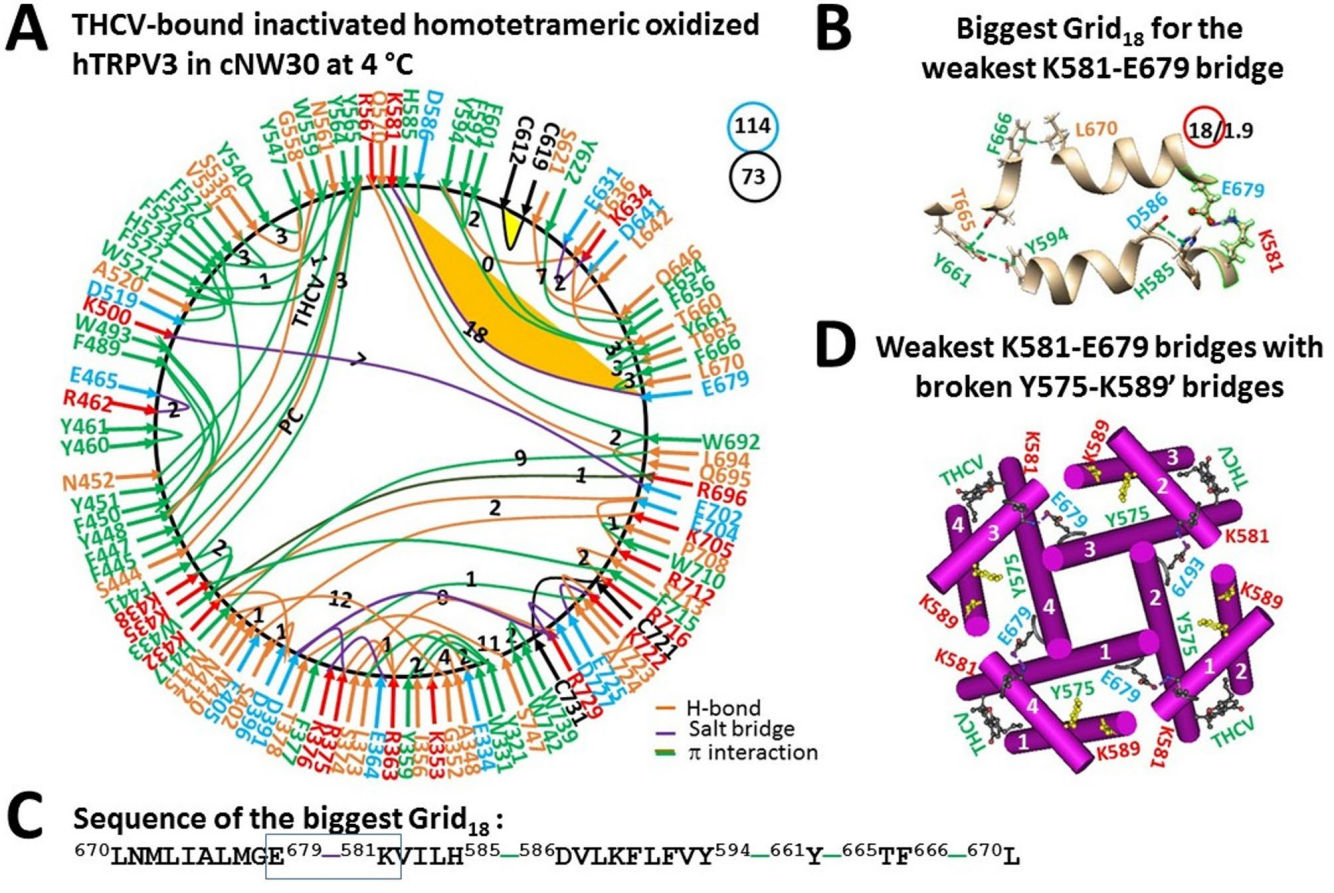
Thermoring structures of inactivated and oxidized hTRPV3 with THCV bound at low temperature. (**A**) The grid-like noncovalently interacting mesh network along the PC-dependent gating pathway of a single subunit of oxidized and inactivated hTRPV3 in the presence of THCV and cNW30 at 4 °C (PDB: 8V6M). Salt bridges, π interactions, and H-bonds between paired amino acid side chains along the PC-dependent gating pathway from K318 to G754 are denoted in purple, green, and orange, respectively. The specific constrained grid sizes necessary to regulate the least-stable noncovalent interactions in the grids are indicated with black numbers. The identified weakest K581-E679 salt bridge in the biggest Grid_18_ is emphasized in orange. The total grid sizes and the total grid size-controlled noncovalent interactions along the PC-dependent gating pathway are displayed in cyan and black circles, respectively. (**B**) The structure of the biggest Grid_18_ with an 18-residue size to regulate the weakest K581-E679 salt bridge at the S4-S5 linker/S6 interface. (**C**) The sequence of the biggest Grid_18_ to control the weakest K581-E679 salt bridge highlighted in the blue box. (**D**) Allosteric** g**ating coupling between the weakest intrasubunit K581-E679 salt bridge and the broken Y575-K589’ swapping interactions near the lower gate in the inactivated state, along with THCV at the vanilloid sites.

In the THCV-induced inactivated state, the least-stable Q346-N394 H-bond at the NLD/ARD interface was disrupted. Meanwhile, the stimulatory D519-R567 H-bond at the S4-S5 linker/VSLD interface was also broken but the inhibitory K581-E679 salt bridge at the S4-S5 linker/S6 interface was present as the least-stable in the biggest Grid_18_ (Fig. 6A)^17^. It had an 18-residue thermoring from L670 to E679, K581, H585, D586, Y594,Y661, T665, F666, and back to L670 (Fig. 6B, C). For 1.9 basic H-bonds to be energetically equivalent to the least-stable K581-E679 bridge, the calculated T_m,th_ to unfold it was about 37 °C, higher than the 32 °C of the open state (Table 1).

Of special note, in addition to the broken Y575-K589’ intersubunit bridges (Fig. 6D), the non-native structure was present in the inactivated state due to the disconnection of the highly conserved D586-T680 and F590-L673 bridges in the normal closed and open states of oxidized mTRPV3 in cNW11 nanodiscs at 42 °C (Fig. 6A)^9^. Furthermore, along the expanded PC-dependent gating pathway from K318 to G754, after reducing the total number of grid sizes and noncovalent interactions from 137 and 82 to 114 and 73, respectively, the systematic thermal instability (T_i_) also decreased from 1.67 to 1.56 (Table 1). Thus, both the increased T_m,th_ and decreased T_i_ indicated that the inactivated state was more stable than the open state in the presence of THCV.

### Swapping Y575-K589’ bridges prime TRPV3 closure before predictable opening

A recent investigation demonstrated that the highly conserved intersubunit interaction between a Tyr residue in the S4-S5 linker from one subunit and an Arg/Lys residue on S5 must be finally disrupted for TRPV1-4 opening^15^. This study further indicated that the swapping Y575-K589’ bridges were formed in the closed state but disrupted in the open state and the subsequent inactivated state (Figs. 4E, 5B, 6D)^17^. Therefore, it is of special interest to test whether disrupting swapping Y575-K589’ bridges can directly open TRPV3.

**Fig. 7:**
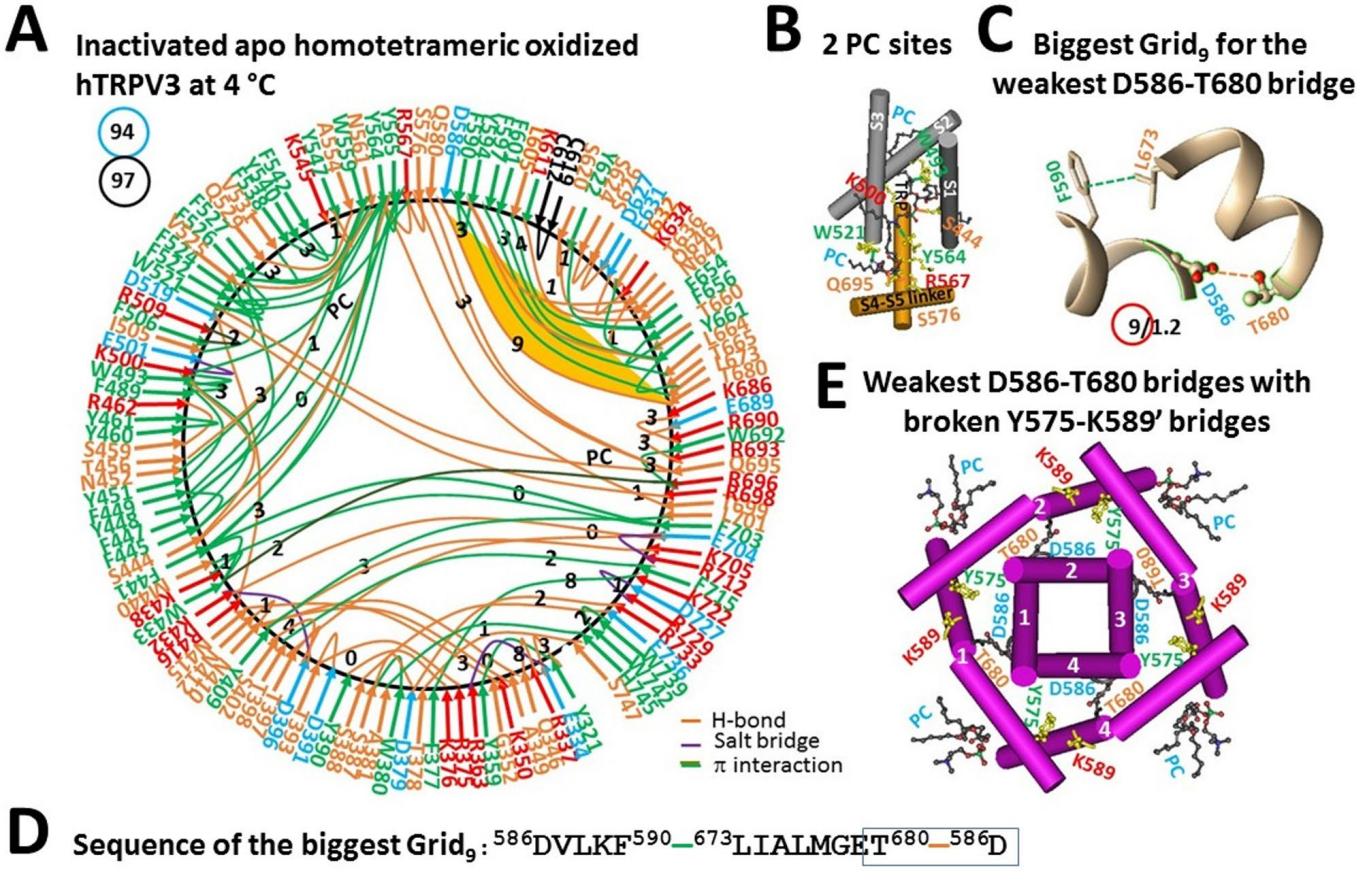
Thermoring structures of apo inactivated and oxidized hTRPV3 without cNW30 at low temperature. (**A**) The grid-like noncovalently interacting mesh network along the PC-dependent gating pathway of a single subunit of oxidized and inactivated hTRPV3 in the absence of THCV and cNW30 at 4 °C (PDB: 8GKA). Salt bridges, π interactions, and H-bonds between paired amino acid side chains along the PC-dependent gating pathway from K318 to G754 are denoted in purple, green, and orange, respectively. The specific constrained grid sizes needed to regulate the least-stable noncovalent interactions in the grids are shown with black numbers. The identified weakest D586-T680 H-bond in the biggest Grid_9_ is emphasized in orange. The total grid sizes and the total grid size-controlled noncovalent interactions along the PC-dependent gating pathway are displayed in cyan and black circles, respectively. (**B**) Two PC sites in the VSLD and at the vanilloid site. (**C)** The structure of the biggest Grid_9_ with a 9-residue size to regulate the wesakest D586-T680 H-bond in the pore domain. (**D**) The sequence of the biggest Grid_9_ to control the weakest D586-T680 H-bond highlighted in the blue box. (**E**) Allosteric** g**ating coupling between the weakest intrasubunit D586-T680 bridges and the broken Y575-K589’ swapping interactions near the lower gate in the inactivated state, along with the PC lipids at the vanilloid sites.

In the absence of cNW30 nanodiscs that prevent membrane diffusive protomer exchange, the PC lipid in the VSLD was linked by S444, W493, K500, and Y564. However, the PC lipid at the vanilloid site of oxidized hTRPV3 was tightly packaged by W521, F522, Y564, R567, S576, Q695 via noncovalent bridges (PDB: 8GKA) (Fig. 7A, B)^35^. When the E467-K545 salt bridge was disconnected, D519 H-bonded not only with R416 in the pre-S1 domain but also with R698 in the TRP domain. Along the similar extended PC-dependent gating pathway from K318 to G754, the total numbers of grid sizes and tertiary noncovalent interactions changed from 143 and 82 to 94 and 97, respectively (Figs. 4A, 7A). Therefore, the systematic thermal instability (T_i_) dramatically decreased from 1.74 to 0.97 (Table1). Furthermore, the highly conserved D586-T680 H-bond in the pore domain, which is necessary for normal TRPV3 gating^9,36^, became the weakest (Fig. 7A, C). It was governed by the biggest Grid_9_ via a thermoring from D586 to F590, L673, T680 and back to D586 (Fig. 7D). When this weakest bridge was energetically equivalent to 1.2 basic H-bond (1.2 kcal/mol), the calculated T_m,th_ was 48 °C, higher than 30 °C of oxidized closed hTRPV3 in the presence of cNW30 nanodiscs (Table 1). Thereafter, both lower T_i_ and higher T_m,th_ suggested a very stable subunit in the initial gating state when the cNW30 nanodiscs were removed.

**Fig. 8:**
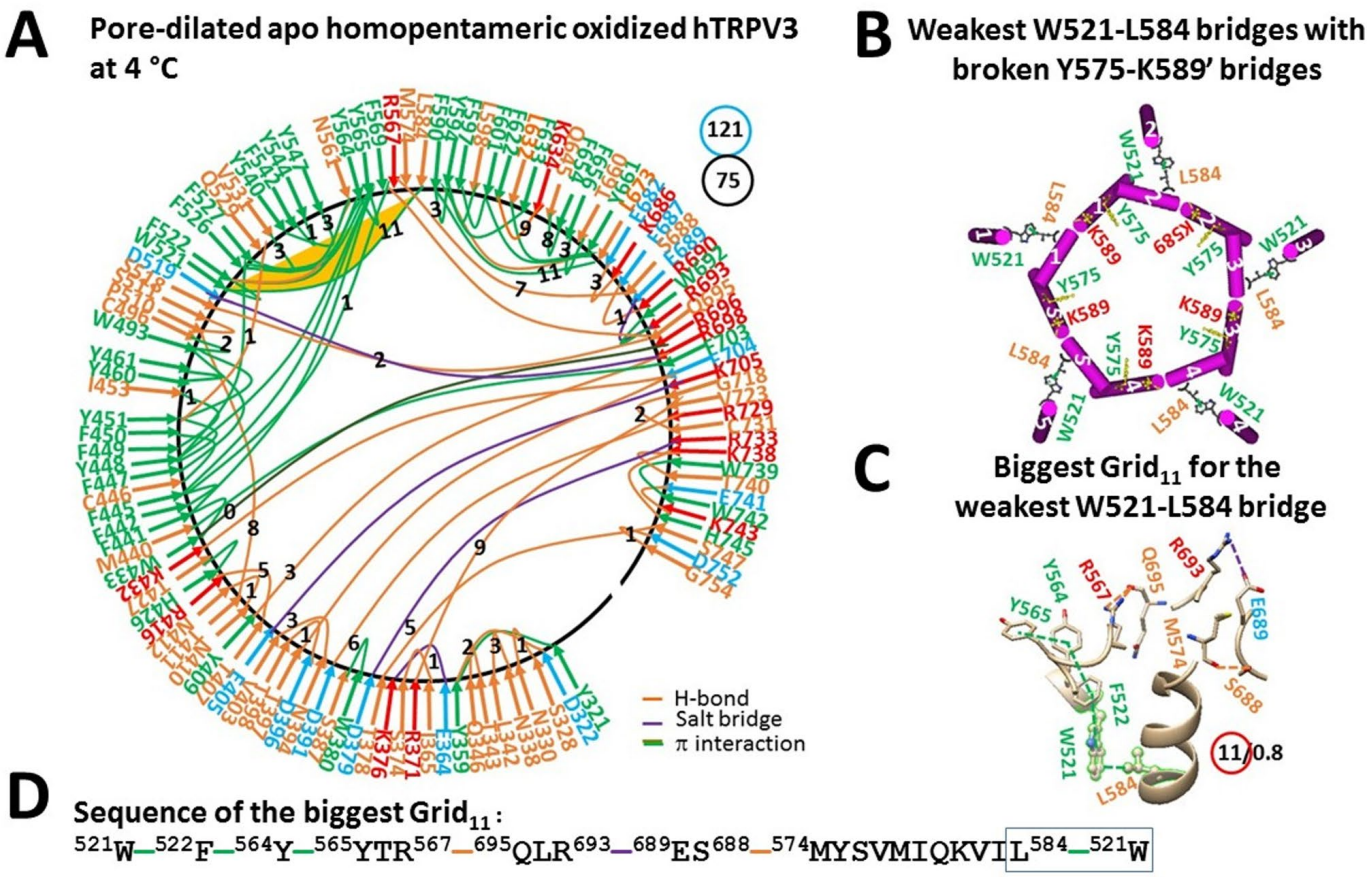
Thermoring structures of pore-dilated and oxidized and pentameric hTRPV3 without THCV at low temperature. (**A**) The grid-like noncovalently interacting mesh network along the PC-dependent gating pathway of a single subunit of oxidized and pore-dilated hTRPV3 in the absence of THCV and cNW30 at 4 °C (PDB: 9DIJ). Salt bridges, π interactions, and H-bonds between paired amino acid side chains along the PC-dependent gating pathway from K318 to G754 are denoted in purple, green, and orange, respectively. The specific constrained grid sizes necessary to regulate the least-stable noncovalent interactions in the grids are indicated with black numbers. The identified weakest W521-L584 π interaction in the biggest Grid_11_ is emphasized in orange. The total grid sizes and the total grid size-controlled noncovalent interactions along the PC-dependent gating pathway are displayed in cyan and black circles, respectively. (**B**) Disrupting D586-T680 bridges coupled the weakest W521-L584 bridges with the broken Y575-K589’ swapping interactions in the pentameric channel for pore dilation. (**C**) The structure of the biggest Grid_11_ with an 11-residue size to regulate the wesakest W521-L584 bridge at the S4-S5 linker/VSLD interface. (**D**) The sequence of the biggest Grid_11_ to control the weakest W521-L584 π interaction highlighted in the blue box.

On the other hand, the critical Y575-K589’ swapping bridges were found to be broken, but the channel pore was still closed (Fig. 7E)^35^. Given that disrupting the weakest D586-T680 bridge disables normal TRPV3 activation^9,36^, the isolated whole hTRPV3 channel could not be opened normally without cNW30 nanodiscs. In other words, the channel was actually inactivated. When the crucial D586-T680 H-bond is broken along with PC release from the vanilloid site (Fig. 8A), this homotetrameric channel partially transitions to the homopentameric one^35,37^. While the weakest W521-L584 bridge was formed between the VSLD and the S4-S5 linker, together with the broken Y575-K589’ bridge (Fig. 8B), favoring pore dilation (Fig. 8A)^37^, it was controlled by the biggest Grid_11_ via a thermoring from W521 to F522, Y564, Y565, R567, Q695, R693, E689, S688, M574, L584 and back to W521 (Fig. 8C, D). When the least-stable W521-L584 bridge was energetically equivalent to 0.8 basic H-bond (0.8 kcal/mol), the T_m,th_ was calculated as 40 °C, lower than the 48 °C of the homotetrameric hTRPV3 (Table 1). Furthermore, along the similar extended PC-dependent gating pathway from K318 to G754, the total numbers of grid sizes and tertiary noncovalent interactions changed from 94 and 97 to 121 and 75, respectively (Figs. 7A, 8A). Therefore, the systematic thermal instability (T_i_) increased from 0.97 to 1.61 (Table 1). In other words, the homopentameric hTRPV3 channel was relatively unstable. Taken together, the swapping Y575-K589’ bridges must remain in the initial resting closed state for normal TRPV3 function. Once they were broken, the whole channel would become unstable and unpredictable, with a smaller fraction of pentamers and a background of lower oligomers^35^, regardless of the initial stable subunits.

## Discussion

The thermosensitive TRPV1-4 channels are well-known for their specific activation thresholds and high temperature sensitivity (Q_10_). The former can be explained by the melting temperature threshold (T_m,th_) of the least-stable noncovalent interaction along the lipid-dependent minimal gating pathway from the pre-S1 domain to the TRP domain^9,13–15^. However, the energy origin of the high Q_10_ is still not well understood when inactivation is involved. Although a single Gibbs–Helmholtz equation has been predicted to link both cold and hot sensations of TRPV1-4 channels for a significant ΔC_p_^10^, cold activation has not been examined for the native construct with inactivation. Since the heat capacity difference (ΔC_p_) underlies heat sensing of TRPV3^9^, cold activation should be detected. In this computational study, symmetric initial cold and heat activations from the same starter were used to further examine this model^10,15^. Quaternary and tertiary thermoring structural analyses demonstrated that different gating states had distinct tertiary thermoring structures for various thermostabilities, leading to temperature-dependent gating pathways of oxidized TRPV3. However, the mirrored thermosensitivity during initial cold and heat activations, once triggered by the PC release from the same active vanilloid site, still confirmed this heat capacity model, regardless of the subsequent inactivation at low temperature. On the contrary, the initial inactivation induced an unpredictable change in the quaternary structure during the pore dilation.

### Gating state-dependent thermostability

Thermodynamic analyses revealed that each gating state of oxidized hTRPV3 in cNW30 nanodiscs at 4 °C exhibited different thermostability. Even with the PC lipid present at the active vanilloid site, the closed state had a calculated T_m,th_ of 30 °C (Table 1). When PC was replaced by THCV at the vanilloid site, the T_m,th_ values of the open and inactivated states increased to 32 °C and 37 °C, respectively (Table 1). In contrast, the closed and open states of oxidized mTRPV3 in cNW11 nanodiscs have T_m,th_ values of 40 °C and 61 °C, respectively^9^. Notably, while the final inactivated state of oxidized hTRPV3 in cNW30 nanodiscs at 4 °C showed the lower systematic thermal instability (T_i_) of 1.56 than the common T_i_ around 1.71 in closed and open states (Table 1), the closed and open states of oxidized mTRPV3 in cNW11 nanodiscs at 42 °C have a lower T_i_ of 1.20^9^. In this regard, these gating state-dependent thermal stabilities at low and high temperatures may lead to asymmetric temperature-dependent gating pathways.

### Asymmetric temperature-dependent gating pathways

**Fig. 9:**
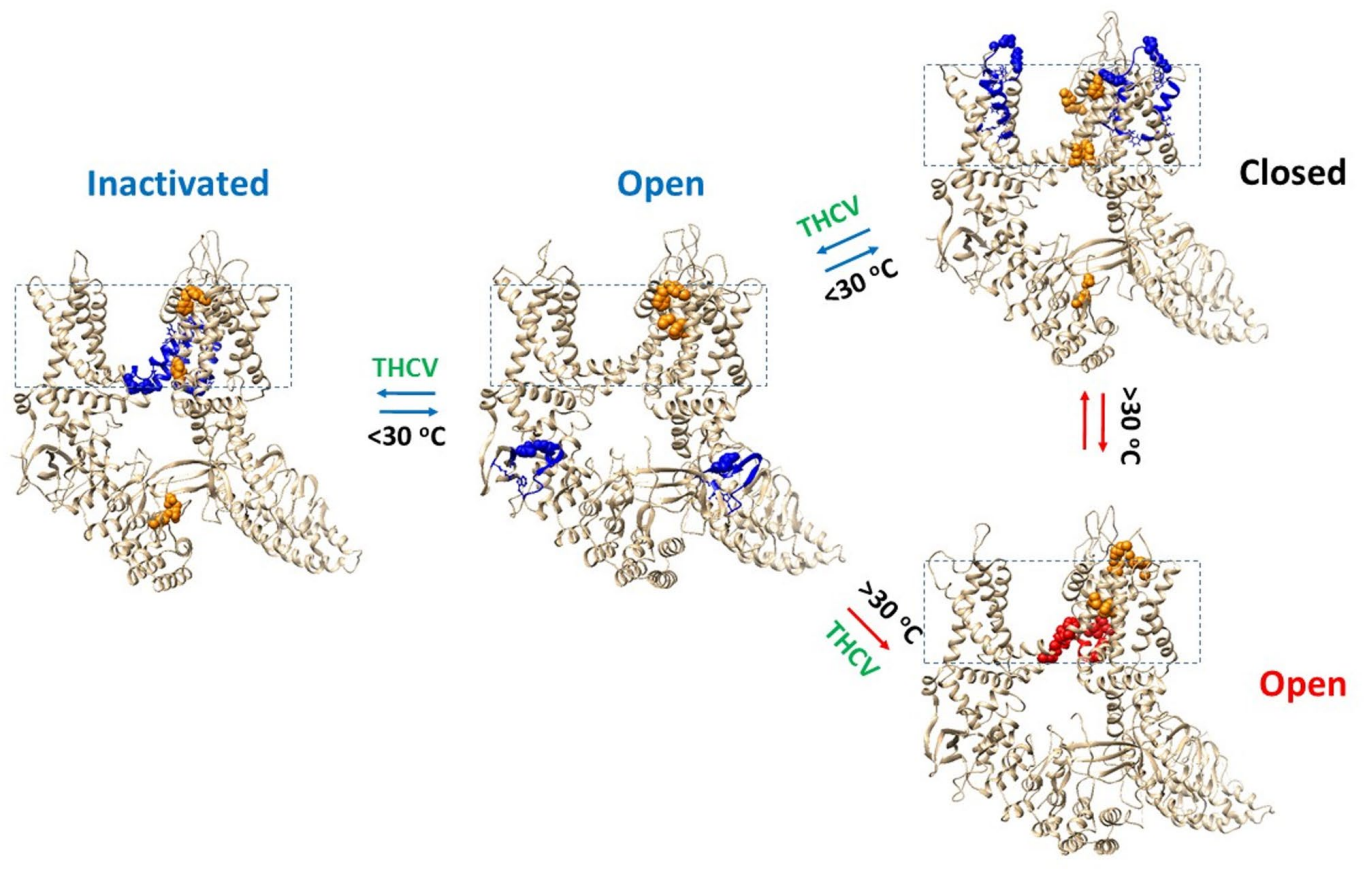
Temperature-dependent gating pathways of oxidized TRPV3. The homo-tetrameric cryo-EM structures of oxidized hTRPV3 in the PC-bound closed state, the THCV bound open and inactivated states at 4 °C (PDB ID, 8V6K, 8V6L and 8V6M, respectively) were used for the model. The homo-tetrameric cryo-EM structure of the oxidized mTRPV3 channel in the open state at 42 °C (PDB ID, 7MIO) was used as a control. The biggest thermorings Grid_17_, Grid_16_ and Grid_18_ in the closed, open and inactivated states below 30 °C are colored blue, respectively. In contrast, the biggest thermoring Grid_9_ in the open state above 30 °C is colored red. All the least-stable noncovalent interactions controlled by those biggest thermorings are indicated by space fill. The intersubunit interactions are colored orange and indicated by space fill.

Oxidized hTRPV3 can remain closed in cNW30 nanodiscs when the temperature is below 30 °C. If the same heat-evoked open state of oxidized mTRPV3 in cNW11 nanodiscs is applied to oxidized hTRPV3 in cNW30 nanodiscs, the release of PC from the active vanilloid site after heat stimuli could disrupt the weakest E467-K545 bridge above 30 °C for channel opening (Fig. 9). In agreement with this proposal, oxidized mTRPV3 can be activated above 30 °C after prolonged heat exposure^3,9,16,32^. Consequently, if a ligand such as THCV displaces the PC lipid from the active vanilloid site, oxidized hTRPV3 could potentially be activated by both heat and cold stimuli through different gating pathways.

Given that oxidized hTRPV3 had a similar T_i_ around 1.71 in the PC-bound closed and THCV-opened states at 4 °C (Table 1), these two gating states may coexist at low temperature as recently proposed^17^. However, their distinct T_m,th_ values were 30 °C and 32 °C, respectively, together with the lower T_i_ of 1.56 and the higher T_m,th_ of 37 °C in the inactivated state (Table 1), may shift the balance from the close state to the open one and then the inactivated one below 30 °C (Fig. 9). On the other hand, oxidized mTRPV3 has the T_m,th_ of 61 °C and the T_i_ of 1.20 (Table 1)^9^. Therefore, an increase in thermostability may allow oxidized TRPV3 in cNW30 nanodiscs to remain open above 30 °C without any inactivation even in the presence of THCV (Fig. 9). In this regard, the structural and functional characterization of ligand or drug binding to a target is necessary at human body temperature. However, the reported cases are still limited^17,38–41^.

### Asymmetric quaternary structures during initial cold and heat activations

Given that homotetrameric oxidized TRPV3 channels had different tertiary thermoring structures in various temperature-dependent gating states (Figs. 3A, 4A, 5A)^9^, it is reasonable that they also exhibited the distinct quaternary structural changes during initial cold and heat activations (Figs. 2–3). Given that oxidized hTRPV3 in cNW30 nanodiscs at 4 °C shared the same R416-D519 and Y594-T636-Y661 bridges with oxidized mTRPV3 in cNW11 nanodiscs at 4 °C in the closed state with PC bound at the same active vanilloid site (Fig. 4A)^9^, it is reasonable that they had the same swapping Y460-F626’ and I637-F666’ π interactions (Figs. 2–3, 9). Furthermore, both closed states exhibited the same swapping K169-D752’ and Y575-K589’ bridges (Fig. 9), in agreement with previous reports^15,33,42^.

Once THCV outcompeted the PC lipid from the active vanilloid site as heat does^9^, although the open state of oxidized hTRPV3 at 4 °C displayed the same stimulatory D519-R567 bridges along with broken R416-D519 and K169-D752’ and Y575-K189’ bridges as the heat-opened oxidized mTRPV3 at 42 °C (Fig. 5A)^9^, the Y460-F625’ swapping bridges in the open state at 4 °C were substituted by the A549-Y650’ ones in the open state at 42 °C, along with the same intersubunit W559-L599’ interactions (Figs. 2, 3 and 9). The same quaternary structure is also present in oxidized and activated hTRPV3 with citronellal at the vanilloid site in the presence of membrane scaffold protein 2N2 (MSP2N2) nanodiscs^41^.

### Symmetric temperature sensitivity during initial cold and heat activations

Since inactivation is the final step of hTRPV3 after cold activation by THCV below 30 °C, a comparison of thermosensitivity between initial cold and heat activations upon the release of PC from the same active vanilloid site could be used to examine the heat capacity model^10,15^. For THCV-induced cold activation of oxidized hTRPV3, a small decrease in total grid sizes from 143 to 137 would result in a lower mean structural cold sensitivity (Ω_10, cold_) of 3.0 (Table 1), similar to the functional heat sensitivity (Q_10, heat_) of 2.32 in mTRPV3^9^. Given that the same rTRPV1 channel exhibits cold and heat activations with symmetric thermosensitivity upon the release of PI from the same active vanilloid site (Fig. 1)^15^, the similar results between cold activation of oxidized hTRPV3 below 30 °C and heat activation of oxidized mTRPV3 above 40 °C upon the release of PC from the same active vanilloid site demonstrate that both cold and hot sensing abilities in either TRPV1 or TRPV3 can be linked and explained by the single Gibbs–Helmholtz equation for a temperature-independent ΔC_p_^10^.

In contrast, the human TRP ankyrin 1 (hTRPA1) channel has been identified as an intrinsic redox state-dependent bidirectional thermosensor with a temperature-dependent gating pathway. Unlike cold activation below 22 °C, its heat activation above 22 °C is accompanied by inactivation or desensitization. More importantly, its initial cold and heat activations are asymmetric^43,44^. Therefore, separate channel structures for cold and heat sensitivity suggest that different quaternary structural rearrangements may be involved in the dual but asymmetric cold and heat sensation of thermosensitive TRPA1 channels to generate a temperature-dependent ΔC_p_^44,45^.

Because *Xenopus* oocytes expressing frog TRPV3 (fTRPV3) also exhibit asymmetric cold activation below 20 °C and heat activation above 50 °C^46,47^, further structural investigations are necessary to determine if the quaternary structures in cold- and heat-evoked open states are different even though they share the same closed fTRPV3 channel.

### Timing of inactivation also triggers various gating pathways

In the heat-evoked inactivation of rTRPV1 from the same pre-open closed state above 43 °C, the R557-E570, D576-T685 and F580-L678 bridges remain conserved. However, the H410-E692/N695 bridges inactivate the rTRPV1, resulting in the absence of swapping Y565-F580’ bridges but the presence of intersubunit R455-E600’ bridges (Fig. 1)^14,15^.

In contrast, in the THCV-induced inactivated state at 4 °C, the same swapping of the K169-D752’ H-bond and the Y460-F625’ π interaction in closed oxidized hTRPV3 was also observed. However, the swapping of the N671-Y575’ π interaction differed from the swapping of the I637-F666’ π interaction (Figs. 2, 3). This discrepancy could be attributed to the presence of the K581-E679 salt bridge in the biggest Grid_18_ and the absence of the D586-T680 and F590-L673 bridges in the inactivated state (Figs. 4A). In any case, the swapping of the Y575-K589’ bridges in hTRPV3 were finally disrupted in the inactivated state (Figs. 6D)^9,15^. Therefore, the initial normal heat or cold activation was not affected. However, the initial inactivation upon breaking the swapping of the Y575-K589’bridges caused hTRPV3 to be pore-dilated along with a significant change in the quaternary structures (Figs. 7E, 8B). Hence, inactivation timing also plays a critical role in regulating the gating pathways of thermosensitive TRP channels.

## Conclusions

Complex protein-lipid interactions make it challenging to understand the heat-sensing mechanism of the thermosensitive TRPV1-4 channels. Based on the Gibbs–Helmholtz equation, symmetric cold and heat activations from the same starter can be used to examine whether hot and cold sensations of thermosensitive TRPV channels can be linked and explained by a single Gibbs–Helmholtz equation for a temperature-independent ΔC_p_. In this study, cryo-EM structures of oxidized TRPV3 with or without tetrahydrocannabivarin (THCV) bound at low and high temperatures were analyzed using a highly-sensitive thermodynamic method. Following the identification of the mirrored temperature sensitivity during initial cold and heat activations by the PC release from the same active vanilloid site, this study supported the heat capacity model, regardless of the involvement of the subsequent inactivation only at low temperature.

## Methods

### Cryo-EM structures used

The cryo-EM 3D structures of the resting closed, activated and inactivated rTRPV1 channels in MSP2N2 nanodiscs at 4 and 48 °C were first sampled as positive controls (PDB ID: 7LP9, model resolution = 2.63 Å; 7RQW/8U3J/8U3L, model resolution = 3.11/2.9/3.7 Å, 7LPE, model resolution = 3.72 Å, and 7LPD, model resolution = 3.55 Å, respectively)^12,30,31^. The cryo-EM 3D structures of the closed and open mTRPV3 channels with the C612-C619 disulfide bond in cNW11 nanodiscs at 4 °C and 42 °C were then examined to show the heat-evoked channel gating transition, respectively  (PDB ID: 7MIM, model resolution = 3.42 Å; 7MIO, model resolution = 3.48 Å, respectively)^16^. In contrast, the cryo-EM 3D structures of the closed, open and inactivated hTRPV3 channels with the C612-C619 disulfide bond in cNW30 nanodiscs at 4 °C were studied to reveal cold-evoked gating transitions upon tetrahydrocannabivarin (THCN) binding (PDB ID: 8V6K, model resolution = 2.46 Å; 8V6L, model resolution = 3.68 Å, and 8V6M, model resolution = 3.63 Å, respectively)^17^. In addition, the cryo-EM 3D structure of the open oxidized hTRPV3 channel with citronellal bound at 4 °C was used as a control for the cold-induced gating transition (PDB ID: 9JE5, model resolution = 3.53 Å respectively)^41^. Finally, the cryo-EM 3D structures of inactivated tetrameric and pore-dilated pentameric hTRPV3 were also employed as controls to indicate the role of the Y575-K589’ swapping interactions in canonical channel gating (PDB ID: 8GKA, model resolution = 2.55 Å; 9DIJ, model resolution = 4.07 Å, respectively)^35,37^.

### Filtering tertiary noncovalent interactions

The stereo-selective and regio-selective inter-domain diagonal and intra-domain lateral noncovalent interactions along the extended PC-dependent gating pathway of hTRPV3 from K318 to G754 were analyzed using UCSF Chimera. The interactions were filtered by the same strict and consistent standard as previously used and confirmed^9,13–15,19–28^. The examined tertiary noncovalent interactions included salt-bridges, lone pair/CH/cation-π interactions and H-bonds between paired amino acid side chains. Specific cutoff distances and interaction angles for the different noncovalent interactions can be found in the online Supporting Information (Table S1, S2, S3, S4 and S5). It is important to note that momentary fluctuation-induced perturbations in tertiary noncovalent interactions during protein dynamics were not considered.

### Mapping thermoring structures using graph theory

The study used the same protocol that had been previously described and validated to map the systematic fluidic grid-like noncovalent interaction mesh network as the thermoring structure^9,13–15,19–28^. In this network, a topological grid was created with nodes representing amino acids and linked nodes representing noncovalent interactions along a single polypeptide chain. Graph theory and the Floyd–Warshall algorithm^48^ were employed to determine the grid size as the shortest round path length to control the least-stable tertiary noncovalent interaction within the grid. The grid size also indicated the minimal number of side chains of free or silent amino acids or atoms that did not participate in any noncovalent interaction within the grid. Uncommon grid sizes were noted in black numbers on the network map alongside the Grid_s_ with an s-residue size. The total numbers of tertiary noncovalent interactions (*N*) and total grid sizes (*S*) along the extended PC-dependent gating pathway of hTRPV3 from K318 to G754 were calculated and displayed in black and cyan circles, respectively, next to the mesh network map for the calculation of systematic thermal instability based on the equation as previously examined^9,13–15,19–28^:

1 $${\mathrm{T}}_{{\mathrm{i}}} = S/N$$

### Calculation of the melting temperature threshold for heat unfolding

The equation used to calculate the melting temperature threshold (T_m,th_) for the heat unfolding of a specific grid has been previously examined^9,13–15,19–28^, and is as follows:

2 $${\mathrm{T}}_{{{\mathrm{m}},{\mathrm{th}}}} (^\circ {\mathrm{C}}) = {34} + \left( {{\text{n }} - { 2}} \right) \times {1}0 + \left( {{2}0{-}{\mathrm{s}}} \right) \times {2}$$

where n represents the total number of basic H-bonds (~ 1 kcal/mol for each) that are energetically equivalent to the least-stable noncovalent interaction controlled by the given grid, and s is the size of the grid that controls the least-stable noncovalent interaction. Therefore, the heat capacity of the grid will increase as the grid size decreases or as the number of equivalent basic H-bonds increases.

### Evaluating the systematic temperature sensitivity

A gating transition of the thermosensitive TRPV1-4 channels is always accompanied by a change in the conformational energy density along the lipid-dependent minimal gating pathway^9,13–15^. Accordingly, along with breaking the weakest bridge to activate hTRPV3 from a closed state within a 10 °C range, based on the assumption that the chemical potential of a grid can be theoretically defined as the maximal potential for equivalent residues in the grid to form the tightest β-hairpin with the smallest loop via noncovalent interactions^49^, the grid-based structural thermo-sensitivity (Ω_10_) of a single ion channel for cold activation could be defined and calculated using the following equations as examined previously^9,13–15^.

3 $$\Omega_{{{1}0}} = \left[ {\left( {{\mathrm{S}}_{{\mathrm{c}}} {-}{\text{ S}}_{{\mathrm{o}}} } \right){\mathrm{E}}/{2}} \right]^{{({\mathrm{Hc}}/{\mathrm{Ho}})}} = \left[ {\left( {{\mathrm{S}}_{{\mathrm{c}}} {-}{\text{ S}}_{{\mathrm{o}}} } \right){\mathrm{E}}/{2}} \right]^{{[({\mathrm{ENc}}/({\mathrm{ENo}})]}} = \left[ {\left( {{\mathrm{S}}_{{\mathrm{c}}} {-}{\text{ S}}_{{\mathrm{o}}} } \right){\mathrm{E}}/{2}} \right]^{{({\mathrm{Nc}}/{\mathrm{No}})}}$$

where along the same defined PC-dependent gating pathway of one subunit, N_c_ and N_o_ represent the total tertiary noncovalent interactions, H_c_ and H_o_ denote the total enthalpy included in them, and S_c_ and S_o_ indicate the total grid sizes in the closed and open states, respectively. The energy intensity of a tertiary noncovalent interaction is denoted by E and is typically 1 kcal/mol^50^. Thus, Ω_10_ factually reflects a thermo-evoked change in the total chemical potential of grids upon a thermo-evoked change in the total enthalpy included in the tertiary noncovalent interactions apparently from a closed state to an open state along the same defined PC-dependent gating pathway of one subunit.

For a convenient comparison, the functional thermo-sensitivity (Q_10_) of a single ion channel for heat activation can be calculated using the following equation:

4 $${\mathrm{Q}}_{{{1}0}} = \left( {{\mathrm{X}}_{{2}} /{\mathrm{X}}_{{1}} } \right)^{{{1}0/({\mathrm{T2}} - {\mathrm{T1}})}}$$

where X_1_ and X_2_ are the relative channel activity obtained at temperatures T1 and T2 (measured in Kelvin), respectively.

## Supplementary Information

Below is the link to the electronic supplementary material.

Supplementary Material 1

## Data Availability

All data generated or analysed during this study are included in this published article and Supporting Information.

## List of symbols

ARD: Ankyrin repeat domain
cryo-EM: Cryogenic electron microscopy
CTD: C-terminal domain
ΔC_p_: Heat capacity difference
Q_10_: Functional thermosensitivity
NLD: N-terminal linker domain
P_o_: Open probability
PI: Phosphatidylinositol
PC: Phosphatidylcholine
PD: Pore domain
Ω_10_: Structural thermosensitivity
T_i_: Systematic thermal instability
T_m,th_: Melting temperature threshold
MSP2N2: Membranescaffoldprotein2n2
RTx: Resiniferatoxin
THCV: Tetrahydrocannabivarin
TRP: Transient receptor potential
TRPV: TRP vanilloid
TRPVi: (I = 1, 2, 3, 4)
fTRPV3: Frog TRPV3
hTRPA1: Human TRP ankyrin 1
hTRPV3: Human TRPV3
mTRPV3: Mouse TRPV3
2-APB: 2-Aminoethoxydiphenylborate
rTRPV1: Rat TRPV1
VSLD: Voltage-sensor-like domain

## References

[1] Caterina, M. J. et al. The capsaicin receptor: A heat-activated ion channel in the pain pathway. *Nature* **389**, 816–824 (1997). 9349813 10.1038/39807

[2] Caterina, M. J., Rosen, T. A., Tominaga, M., Brake, A. J. & Julius, D. A capsaicin-receptor homologue with a high threshold for noxious heat. *Nature* **398**, 436–441 (1999). 10201375 10.1038/18906

[3] Peier, A. M. et al. A heat-sensitive TRP channel expressed in keratinocytes. *Science* **296**, 2046–2049 (2002). 12016205 10.1126/science.1073140

[4] Smith, G. D. et al. TRPV3 is a temperature-sensitive vanilloid receptor-like protein. *Nature* **418**, 186–190 (2002). 12077606 10.1038/nature00894

[5] Xu, H. et al. TRPV3 is a calcium-permeable temperature-sensitive cation channel. *Nature* **418**, 181–186 (2002). 12077604 10.1038/nature00882

[6] Guler, A. D. et al. Heat-evoked activation of the ion channel, TRPV4. *J. Neurosci.* **22**, 6408–6414 (2002). 12151520 10.1523/JNEUROSCI.22-15-06408.2002 PMC6758176

[7] Chung, M. K., Lee, H. & Caterina, M. J. Warm temperatures activate TRPV4 in mouse 308 keratinocytes. *J. Biol. Chem.* **278**, 32037–32046 (2003). 12783886 10.1074/jbc.M303251200

[8] Yao, J., Liu, B. & Qin, F. Modular thermal sensors in temperature-gated transient receptor potential (TRP) channels. *Proc. Natl. Acad. Sci.* **108**, 11109–11114 (2011). 21690353 10.1073/pnas.1105196108 PMC3131340

[9] Wang, G. Thermoring basis for the TRPV3 bio-thermometer. *Sci. Rep.* **13**, 21594 (2023). 38062125 10.1038/s41598-023-47100-0 PMC10703924

[10] Clapham, D. E. & Miller, C. A thermodynamic framework for understanding temperature sensing by transient receptor potential (TRP) channels. *Proc. Natl. Acad. Sci. U.S.A.* **108**, 19492–19497 (2011). 22109551 10.1073/pnas.1117485108 PMC3241781

[11] Babu, C. R., Hilser, V. J. & Wand, A. J. Direct access to the cooperative substructure of proteins and the protein ensemble via cold denaturation. *Nat. Struct. Mol. Biol.* **11**, 352–357 (2004). 14990997 10.1038/nsmb739

[12] Kwon, D. H. et al. Heat-dependent opening of TRPV1 in the presence of capsaicin. *Nat Struct Mol Biol.* **28**, 554–563 (2021). 34239123 10.1038/s41594-021-00616-3 PMC8335751

[13] Wang, G. Thermoring-based heat activation switches in the TRPV1 biothermometer. *Int. J. Biol. Macromol.* **248**, 125915 (2023). 37481175 10.1016/j.ijbiomac.2023.125915

[14] Wang, G. Thermoring basis for heat unfolding-induced inactivation in TRPV1. *Nat. Sci.* **4**, e20240008 (2024).

[15] Wang, G. Pathway-dependent cold activation of heat-responsive TRPV channels. *Sci. Rep.* **15**, 45041 (2025). 41326491 10.1038/s41598-025-29524-y PMC12749286

[16] Nadezhdin, K. D. et al. Structural mechanism of heat-induced opening of a temperature-sensitive TRP channel. *Nat. Struct. Mol. Biol* **28**, 564–572 (2021). 34239124 10.1038/s41594-021-00615-4 PMC8283911

[17] Nadezhdin, K. D. et al. TRPV3 activation by different agonists accompanied by lipid dissociation from the vanilloid site. *Sci Adv.* **10**, eadn2453 (2024). 38691614 10.1126/sciadv.adn2453 PMC11062575

[18] Yao, J., Liu, B. & Qin, F. Pore turret of thermal TRP channels is not essential for temperature sensing. *Proc. Natl. Acad. Sci. U. S. A.* **107**, E125 (2010). 20660307 10.1073/pnas.1008272107 PMC2922611

[19] Wang, G. The network basis for the structural thermostability and the functional thermoactivity of aldolase B. *Molecules* **28**, 1850 (2023). 36838836 10.3390/molecules28041850 PMC9959246

[20] Wang, G. Network basis for the heat-adapted structural thermostability of bacterial class II fructose bisphosphate aldolase. *ACS Omega* **8**, 17731–17739 (2023). 37251155 10.1021/acsomega.3c00473 PMC10210171

[21] Wang, G. Thermal ring-based heat switches in hyperthermophilic class II bacterial fructose aldolase. *ACS Omega* **8**, 24624–24634 (2023). 37457467 10.1021/acsomega.3c03001 PMC10339327

[22] Wang, G. Phosphatidylinositol-4,5-biphosphate (PIP_2_)-dependent thermoring basis for cold-sensing of the transient receptor potential melastatin-8 (TRPM8) biothermometer. *Physchem* **4**, 106–119 (2024).

[23] Wang, G. ATP-dependen thermoring basis for the heat unfolding of the first nucleotide-binding domain isolated from CFTR. *Nat. Sci.* **5**, e70007 (2025).

[24] Wang, G. Trikafta rescues F508del-CFTR by tightening specific phosphorylation-dependent interdomain interactions. *Nat. Sci.* **6**, e70009 (2025).

[25] Wang, G. Thermoring basis for thermo-gated TRPV2. Res Sq [Preprint]. rs.3.rs-6049325 (2025).

[26] Wang, G. Thermodynamic coupling between folding correctors and the first of dimerized nucleotide binding domains in CFTR. *ACS. Bio. Med. Chem. Au.* **5**, 593–601 (2025). 40860030 10.1021/acsbiomedchemau.5c00014 PMC12371502

[27] Wang, G. Trikafta restores thermodynamic coupling between two nucleotide binding domains for potentiating CFTR activity. *Biomed. Pharmacother.* **194**, 118936 (2026). 41496342 10.1016/j.biopha.2025.118936

[28] Wang, G. Thermoring basis for the proton-driven heat activation of a cation-selective channel in myriapods. *Sci. Rep.* **16**, 1949 (2026). 10.1038/s41598-025-31732-5 PMC12804983 41398032

[29] Teng, J., Anishkin, A., Kung, C. & Blount, P. Human mutations highlight an intersubunit cation-π bond that stabilizes the closed but not open or inactivated states of TRPV channels. *Proc. Natl. Acad. Sci. U. S. A.* **116**, 9410–9416 (2019). 31010928 10.1073/pnas.1820673116 PMC6511060

[30] Kwon, D. H. et al. Vanilloid-dependent TRPV1 opening trajectory from cryoEM ensemble analysis. *Nat. Commun.* **13**, 2874 (2022). 35610228 10.1038/s41467-022-30602-2 PMC9130279

[31] Arnold, W. R. et al. Structural basis of TRPV1 modulation by endogenous bioactive lipids. *Nat. Struct. Mol. Biol.* **31**, 1377–1385 (2024). 38698206 10.1038/s41594-024-01299-2 PMC11402599

[32] Liu, B. & Qin, F. Single-residue molecular switch for high-temperature dependence of vanilloid receptor TRPV3. *Proc. Natl. Acad. Sci. U. S. A.* **114**, 1589–1594 (2017). 28154143 10.1073/pnas.1615304114 PMC5321025

[33] Deng, Z. et al. Gating of human TRPV3 in a lipid bilayer. *Nat. Struct. Mol. Biol.* **27**, 635–644 (2020). 32572252 10.1038/s41594-020-0428-2 PMC7354234

[34] Kim, S. B., Palmer, J. C. & Debenedetti, P. G. Computational investigation of cold denaturation in the Trp-cage miniprotein. *Proc. Natl. Acad. Sci. U. S. A.* **113**, 8991–8996 (2016). 27457961 10.1073/pnas.1607500113 PMC4987839

[35] Lansky, S. et al. A pentameric TRPV3 channel with a dilated pore. *Nature* **621**, 206–214 (2023). 37648856 10.1038/s41586-023-06470-1 PMC10584365

[36] Shimada, H. et al. The structure of lipid nanodisc-reconstituted TRPV3 reveals the gating mechanism. *Nat. Struct. Mol. Biol.* **27**, 645–652 (2020). 32572254 10.1038/s41594-020-0439-z

[37] Lansky, S., Wang, Z., Clarke, O. B., Chipot, C. & Scheuring, S. Structural dynamics and permeability of the TRPV3 pentamer. *Nat. Commun.* **16**, 4520 (2025). 40374654 10.1038/s41467-025-59798-9 PMC12081643

[38] Bansia, H. et al. Investigating gating mechanisms of ion channels using temperature-resolved cryoEM. *Microsc. Microanal.* **27**, 1690–1694 (2021). 37644957 10.1017/s1431927621006206 PMC10464605

[39] Hu, J. et al. Physiological temperature drives TRPM4 ligand recognition and gating. *Nature* **630**, 509–515 (2024). 38750366 10.1038/s41586-024-07436-7 PMC11168932

[40] Kumar Mondal, A., Carrillo, E., Jayaraman, V. & Twomey, E. C. Glutamate gating of AMPA-subtype iGluRs at physiological temperatures. *Nature* **641**, 788–796 (2025). 40140570 10.1038/s41586-025-08770-0 PMC12074995

[41] Li, Y. et al. Plant essential oil targets TRPV3 for skin renewal and structural mechanism of action. *Nat Commun.* **16**, 2728 (2025). 40108208 10.1038/s41467-025-58033-9 PMC11923102

[42] Zubcevic, L., Borschel, W. F., Hsu, A. L., Borgnia, M. J. & Lee, S. Y. Regulatory switch at the cytoplasmic interface controls TRPV channel gating. *Elife* **8**, e47746 (2019). 31070581 10.7554/eLife.47746 PMC6538378

[43] Moparthi, L. et al. Human TRPA1 is a heat sensor displaying intrinsic U-shaped thermosensitivity. *Sci. Rep.* **6**, 28763 (2016). 27349477 10.1038/srep28763 PMC4923899

[44] Moparthi, L. et al. The human TRPA1 intrinsic cold and heat sensitivity involves separate channel structures beyond the N-ARD domain. *Nat. Commun.* **13**, 6113 (2022). 36253390 10.1038/s41467-022-33876-8 PMC9576766

[45] Yeh, F., Jara-Oseguera, A. & Aldrich, R. W. Implications of a temperature-dependent heat capacity for temperature-gated ion channels. *Proc. Natl. Acad. Sci. U. S. A.* **120**, e2301528120 (2023). 37279277 10.1073/pnas.2301528120 PMC10268252

[46] Saito, S., Fukuta, N., Shingai, R. & Tominaga, M. Evolution of vertebrate transient receptor potential vanilloid 3 channels: Opposite temperature sensitivity between mammals and Western clawed frogs. *PLoS Genet.* **7**, e1002041 (2011). 21490957 10.1371/journal.pgen.1002041 PMC3072374

[47] Liu, B. & Qin, F. The *Xenopus tropicalis* orthologue of TRPV3 is heat sensitive. *J. Gen. Physiol.* **146**, 411–421 (2015). 26458875 10.1085/jgp.201511454 PMC4621749

[48] Floyd, R. W. Algorithm-97: Shortest path. *Commun. ACM* **5**, 345–345 (1962).

[49] Kiehna, S. E. & Waters, M. L. Sequence dependence of β-hairpin structure: Comparison of a salt bridge and an aromatic interaction. *Protein Sci.* **12**, 2657–2667 (2003). 14627727 10.1110/ps.03215403 PMC2366975

[50] Neel, A. J., Hilton, M. J., Sigman, M. S. & Toste, F. D. Exploiting non-covalent π interactions for catalyst design. *Nature* **543**, 637–646 (2017). 28358089 10.1038/nature21701 PMC5907483

