# Supplementary material for: Temperature-dependent gating pathways in TRPV3
Source: Sci Rep. 2026 Mar 26;16:15030. doi: 10.1038/s41598-026-44194-0 (PMC13172410; doi:10.1038/s41598-026-44194-0)
Supplement: Supplementary file 1 — Supplementary Material 1 [file 41598_2026_44194_MOESM1_ESM.pdf]

Supporting Information for

Temperature-dependent gating pathways in TRPV3

Guangyu Wang 1, 2*

[1]Department of Physiology and Membrane Biology, University of California School of Medicine, Davis, CA, USA

[2]Department of Drug Research and Development, Institute of Biophysical Medico-chemistry, Reno, NV, USA

* Correspondence: gary.wang10@gmail.com

This Supporting Information includes:

Tables S1, S2, S3, S4 and S5.

Table S1. Tertiary noncovalent interactions along the PC-dependent gating pathway from K318 to G754 in each subunit of oxidized hTRPV3 with PC bound in the closed state at 4 °C (PDB ID, 8V6K).

**Noncovalent interaction**	**Cut-off distance**	**Linked residues**
Salt bridge	3.2-4 Å	E334-R363, E364-K376, E467-K545, K500-E501, E689-R693, D727-R729
H-bond	<3.9 Å donor-H-acceptor <60°	A349/K350-N410-N412, R363-L373, E367-R369, S374-R729, D379-R733, G383-H745, **D396-K432**, **E405-K705**, R416-D519, K432-E704, S444-PC-W493, S444-PC-H523, Y448-G558/N561, **T456-W559**, E466-I469, Q483-R487, Q529-A554, Y564-R567, Q580-Q695, D586-T680, **Y594-T636,** S620-Y622**,** D641-L642, N643-Q645/Q646, E679-N683-K686, L707-R712, E719-R733, K722-E725, K743-T744
π–π interaction	2.65–6.5 Å	Y321-Y359**, W433-F441-Y565**, F449-**F445-Y565**, **F447**-W493, **Y448-F526**/**Y565**, F449-F450/W559, **Y451-W493**, Y460-Y461, R493-PC-K500-PC-H523, **W521-F522-Y564**, **F526-Y564**, **Y540-Y547**, **Y564-Y565**, **Y661-Y594,** F597-F601**, F601-Y661**, **Y622-F654,** W739-W742
cation–π interaction	<6.0 Å	**W433-R696**
CH_3/CH–π interaction	2.65-3.01 Å	F377-V723**,** W380-S387**, W433-K438**, M440-F703, F449-I453, **N452-W559**, **F527-V531**, Q570-W692, F590-L673, F597-L664, F625-V629, W692-R696, W710-L711
Lone pair–π interaction	3-3.7 Å	**H417-E418**, W521-PC, F656-T660, **T665-Y661**

Note: Bold interactions were conserved in closed and open and inactivated states.

Table S2. Tertiary noncovalent interactions along the PC-dependent gating pathway from K318 to G754 in each subunit of oxidized hTRPV3 with THCN bound in the open state at 4 °C (PDV ID, 8V6L).

**Noncovalent interaction**	**Cut-off distance**	**Linked residues**
Salt bridge	3.2-4 Å	E364-K366, K376-E736, D379-R733, D391-R729, D396-R716-E704, K500-E702, E631-K634, E689-R693, E709-R712, E736-K738
H-bond	<3.9 Å donor-H-acceptor <60°	E334-R363, Q346-N394, S372-D727, S374-R729, K376-S388, Y390-E736, T399-D400, E405-K705, T411-D512, S444-Y565, Y451-Q529, **T456-W559**, R464-E467, P470-Y547, T476-Q483, D519-R567, S536-Y547, K545-E546, R567-W692, **D586-T680**, **Y594-T636-Y661,** D618-S620, L632-T636/Y661, L707-R712
π–π interaction	2.65–6.5 Å	Y321-Y359, H426-H430, **W433-F441-Y565**, F441-F703, **F445-Y565**, **F447-W493**-F489, **Y448-Y451, Y448-F526, Y448-Y565,** F449-F450/W559, **Y451-W493**/F489, H471-**Y540-Y547**-H471, **W521-F522-Y564**, W521-THCN-N561, **F526-Y564**, F542-Y544**,** **Y564-Y565**, F590-Y594, **F601-Y661**, **Y622-F654**, W739-W742
cation–π interaction	<6.0 Å	W331-R729, **H417-R690, W433- R696**
CH_3/CH–π interaction	2.65-3.01 Å	K318-Y359, F377-V723, **H417-E418**, **W433-K438**, F436-F715, F449-I453, **N452-W559**, **F527-V531,** Q570-W692, F590-L673, F597-L664, **F625-V629, T665-Y661**
Lone pair–π interaction	3-3.7 Å	W380-S387

Note: Bold interactions were conserved in closed and open and inactivated states.

Table S3. Tertiary noncovalent interactions along the PC-dependent gating pathway from K318 to G754 in each subunit of oxidized hTRPV3 with THCN bound in the inactivated at 4 °C (PDV ID, 8V6M).

Noncovalent interaction	Cut-off distance	Linked residues
Salt bridge	3.2-4 Å	E364-K376, R375-D391-R729, R462-E465, K500-E702, K581-E679, K722-E725, D727-R729, E631-K634
H-bond	<3.9 Å donor-H-acceptor <60°	E334-R363, A348-K353, G352-N415, R363-L373/R375, S374-R729, T378-S747, D396-S402, **D396-K432**, **E405-K705**, N410-N412, K432-E704, S444-PC-Y565, Y448-G558, S536-Y547, R567-Q695, **Y594-T636-Y661,** S621-Q646**,** D641-L642, E704-R712, S713-R716, K722-A724
π–π interaction	2.65–6.5 Å	Y321-Y359, **W433-F441-Y565**, **F445-Y565**, **F447-F489/W493**, **Y448-F526**, **Y448-Y565**, F450-F489, **Y451-W493**, Y460-Y461, **W521-F522-Y564**, W521-THCV-N561, **F526-Y564**, **Y540-Y547**, **Y564-Y565**, Y594-Y661, F597-**F601-Y661**, **Y622-F654,**W739-W742
cation–π interaction	<6.0 Å	**W433-R696**
CH_3/CH–π interaction	2.65-3.01 Å	Y321-I356, W331-I373, F377-V723, H417-L694, **W433-K438**, K435-F715, **N452-W559**, A520-F524, **F527-V531,** Q570-W692-R696, F666-L670, P708-W710
Lone pair–π interaction	3-3.7 Å	**H417-E418**, D519-H523, H585-D586, F656-T660, **T665-Y661**

Note: Bold interactions were conserved in closed and open and inactivated states.

Table S4. Tertiary noncovalent interactions along the PC-dependent gating pathway from K318 to G754 in each subunit of oxidized hTRPV3 with PC bound in the inactivated state at 4 °C (PDB ID, 8GKA).

Noncovalent interaction	Cut-off distance	Linked residues
Salt bridge	3.2-4 Å	E334-R363, D396-K432, K500-E501, E704-R712, D727-R729
H-bond	<3.9 Å donor-H-acceptor <60°	Y321-E334, R337-Q346, A349/K350-N410, G352-N415, R375-T393, K376-Y390, **T378-S747**, D379-S387/R733, S388-E736, D391-T393, D396-T397/S402, T397-E704, Y409-K705, **N410-N412**, R416-D519, K432-E704, S444-PC-W493, S444-PC-Y564, T456-W559, S459-R462, D519-R698, W521-PC-Q695, V525-N561, Q529-A554, K545-Y547, Y564-R567, R567-PC-S576, S576-PC-Q695, Q580-Q695, D586-T680, Y594-T636-**Y661-Y594**, K611-N647, S620-Y622/Q646, S624-S626-D627, E631-K634, K686-R690, **E689-R693**
π–π interaction	2.65–6.5 Å	Y321-Y359, W433-F441-Y564/Y565/F703, F445-Y448/Y565, **F447-W493-Y451**, **Y448-F526/Y565**, F449-W559, **Y460-Y461**, W493-PC-K500, K500-PC-Y564, **W521-F522-Y564**, W521-PC-R567, F522-**F526-Y564**, F538-**F542**, **Y540-Y547**, **Y564-Y565**, **F601-Y661**, Y622-F654, W739-W742
cation–π interaction	<6.0 Å	F506-R509, **R696-W433**
CH_3/CH–π interaction	2.65-3.01 Å	F377-K722, A381-H745, T398-F715, Y409-L701, W433-K438, **M440-F703**, **N452-W559**, F489-W493, I505-F524, **W521-V525**, **F527-V531**, F590-Y594/L673, F597-L664, L605-F654, **L632-Y661,** W692-**R696**
Lone pair–π interaction	3-3.7 Å	**W380-S387**, **F656-T660**, T665-Y661

Note: Bold interactions were conserved in inactivated and pore-dilated states.

Table S5. Tertiary noncovalent interactions along the PC-dependent gating pathway from K318 to G754 in each subunit of oxidized hTRPV3 with PC bound in the pore-dilated state at 4 °C (PDB ID, 9DIJ).

Noncovalent interaction	Cut-off distance	Linked residues
Salt bridge	3.2-4 Å	E364-K376, D379-K733, **E405-K705**, D519-R698, **E689-R693**
H-bond	<3.9 Å donor-H-acceptor <60°	D322-Y359, S328-N330, N338-L342, T343-Q346, I365-S374, R371-V723, **T378-S747**, D391-R729, N394-V403, D396-T398, **T397-E704**, T407-R416, Y409-R698, **N410-N412**, T411-P510-S511, **K432-E704**, Y451-Q529, S518-Q695, F522-N561, R567-Q695, M574-S688, **Y594-Y661**, Y622-K634, E682-K686, E687-R690, G718-C731, K738-T740/H745, E741-K743,D752-G754
π–π interaction	2.65–6.5 Å	Y321-Y359, **W433-F441-Y565**, F442-F445, **F445-Y565**/F569, C446-F450, **F447-W493-Y451**, Y448-Y451, **Y448-F526/Y565**, F449-I453, **Y460-Y461**, **W521-F522-Y564**, **F526-Y564**, **Y540-Y547**, **F542-Y544, Y564-Y565**, F597-**F601-Y661**, F594-F633, **W739-W742**
cation–π interaction	<6.0 Å	**R696-W433,** W692-R693
CH_3/CH–π interaction	2.65-3.01 Å	**M440-F703**, W493-C496, W521-L584, **F527-V531, F590-Y594/L673**, F594-L598, **L632-Y661**,
Lone pair–π interaction	3-3.7 Å	**W380-S387**, H426-T427, F526-N561, Q645-F654, **F656-T660**

Note: Bold interactions were conserved in inactivated and pore-dilated states.
